# Supplementary figures and images for: Facial shape and allometry quantitative trait locus intervals in the Diversity Outbred mouse are enriched for known skeletal and facial development genes
Source: PLoS One. 2020 Jun 5;15(6):e0233377. doi: 10.1371/journal.pone.0233377 (PMC7274373; doi:10.1371/journal.pone.0233377)

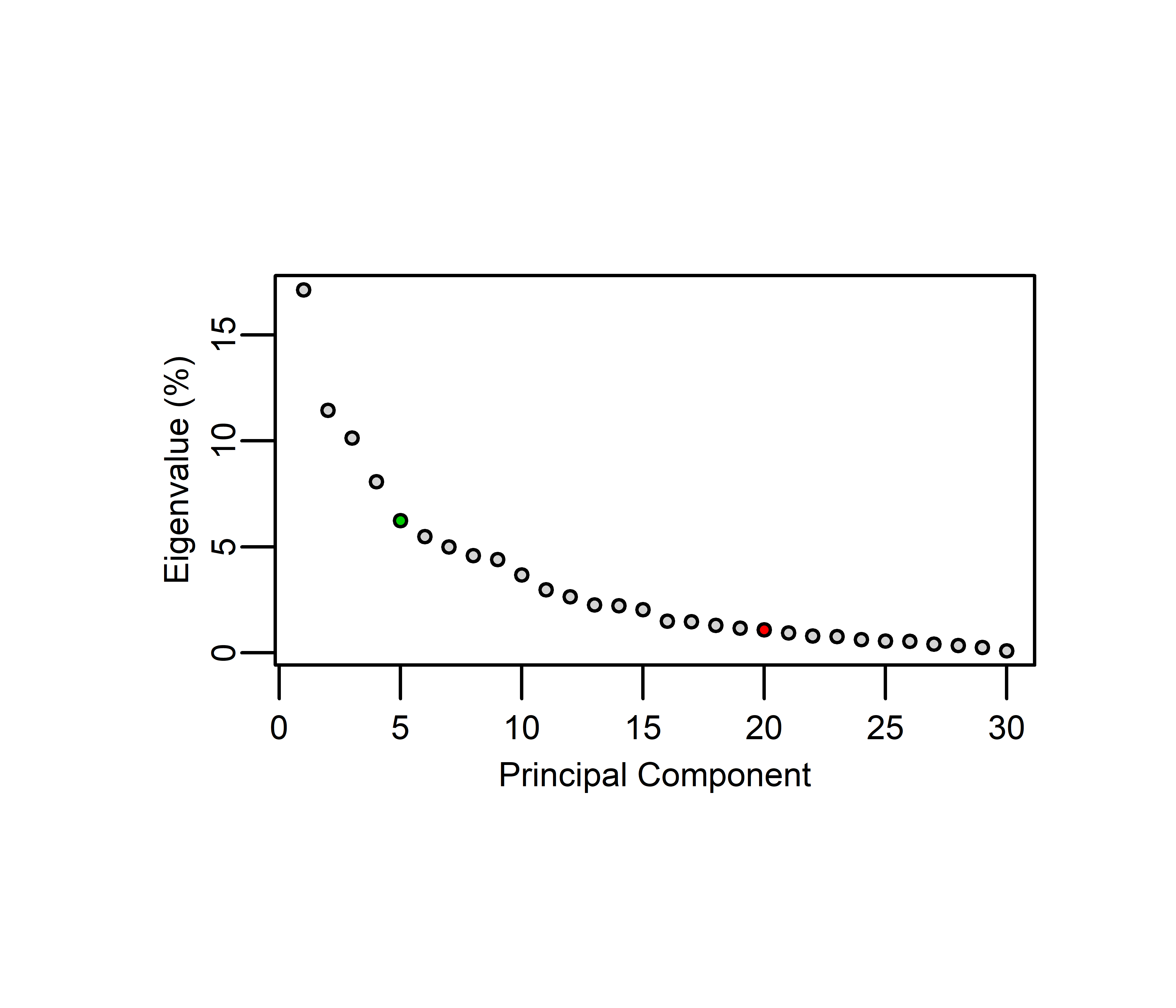

Supplement: S1 Fig — Percentage of total variation explained for each shape principal component. We performed genome scans for all PCs explaining more than 1% of shape variation (red point identifies cutoff at PC 20) and subjected the first five PCs (green point identifies PC 5 floor) to in-depth analysis. (PNG) [file pone.0233377.s001.png]

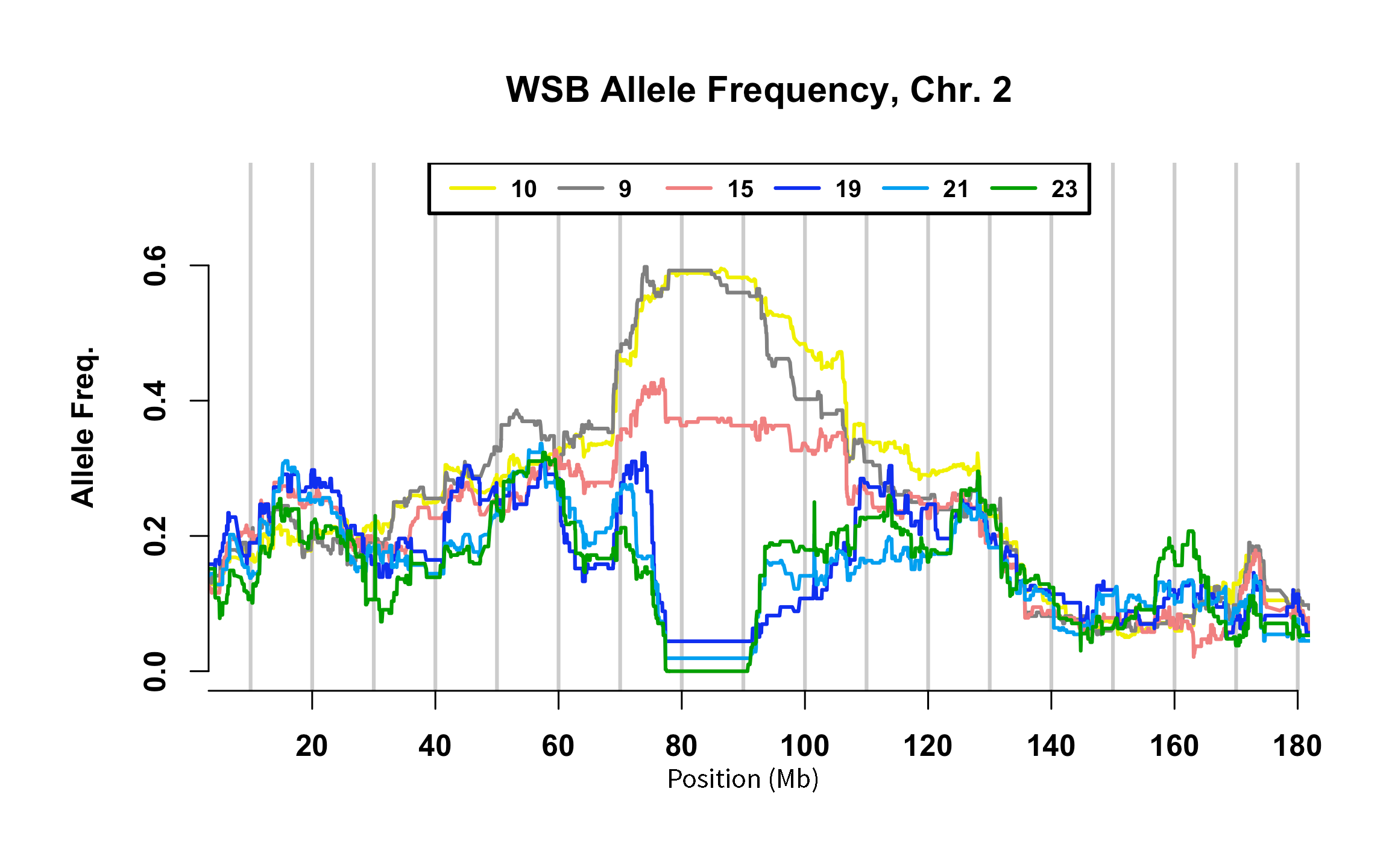

Supplement: S2 Fig — Sample-wide WSB allele frequency on chromosome 2 by DO generation. The high WSB allele frequency in earlier generations reflects meiotic drive for WSB at the R2d2 locus. Declining frequencies in later generations reflect the systematic purge of WSB alleles from the DO population. (PNG) [file pone.0233377.s002.png]
