# Supplementary material for: Facial shape and allometry quantitative trait locus intervals in the Diversity Outbred mouse are enriched for known skeletal and facial development genes
Source: PLoS One. 2020 Jun 5;15(6):e0233377. doi: 10.1371/journal.pone.0233377 (PMC7274373; doi:10.1371/journal.pone.0233377)
Supplement: S1 Table — LOD peaks and support intervals for all QTL on PCs 1–20. (PDF) [file pone.0233377.s003.pdf]

PC	Chr.	Position cM	LOD threshold	LOD	Position Mbp	CI Mbp Low	CI Mbp High	CI Widths
PC1	1	26.650	7.4316	7.47	51.92662	51.84963	53.29940	1.44978
PC1	6	46.261	7.4316	10.43	99.63722	98.87835	100.25880	1.38045
PC1	7	35.436	7.4316	11.59	65.97085	56.39222	66.70274	10.31051
PC2	6	2.227	7.4068	8.64	5.70473	3.37161	6.03770	2.66609
PC2	14	12.035	7.4068	7.57	22.05840	21.51874	31.93686	10.41812
PC3	12	3.887	7.4826	12.93	8.44203	8.23271	9.83742	1.60471
PC3	12	7.752	7.4826	7.55	16.28680	15.57275	17.40083	1.82808
PC4	17	22.576	7.3454	8.33	45.63977	44.73754	48.21456	3.47702
PC6	7	77.768	7.5869	8.54	133.96430	133.15529	134.45830	1.30301
PC7	3	19.668	7.3528	8.39	41.09667	39.97819	46.49435	6.51615
PC7	17	9.119	7.3528	7.53	15.19797	12.69950	28.17045	15.47096
PC7	18	29.197	7.3528	7.80	54.45076	39.71517	54.57177	14.85659
PC9	8	33.131	7.4073	7.75	66.36843	61.78359	67.14575	5.36216
PC9	8	33.547	7.4073	7.90	68.11571	67.49771	68.73613	1.23842
PC9	8	40.818	7.4073	7.76	84.29230	82.44561	117.36733	34.92172
PC9	11	62.326	7.4073	7.92	98.59369	82.09133	106.60060	24.50927
PC10	15	16.562	7.4444	8.45	42.43605	40.16640	52.33545	12.16905
PC10	16	34.119	7.4444	8.00	56.65842	55.36893	69.57857	14.20965
PC12	3	18.610	7.2631	7.71	38.47037	37.94061	39.68852	1.74791
PC12	15	25.967	7.2631	7.27	61.61672	58.25213	65.30036	7.04823
PC13	15	24.482	7.3721	7.56	58.21462	57.77819	59.19820	1.42001
PC13	15	26.147	7.3721	8.24	61.86418	60.81292	63.76806	2.95514
PC14	17	56.011	7.4216	7.50	85.72647	43.53622	86.18045	42.64424
PC15	4	83.406	7.3487	7.64	152.85288	152.44320	153.63442	1.19122
PC15	10	57.592	7.3487	7.75	110.39634	107.71115	110.44517	2.73402
PC15	X	45.660	7.3487	8.75	102.71168	102.14335	104.46776	2.32441
PC16	12	22.220	7.4643	8.32	52.76945	49.79955	53.96553	4.16598
PC17	1	54.938	7.3876	7.67	126.11498	121.31522	128.49124	7.17602
PC17	4	40.835	7.3876	9.63	86.99807	83.20196	87.04323	3.84127
PC17	7	74.072	7.3876	8.06	131.48256	131.28649	131.94025	0.65376
PC17	7	81.000	7.3876	7.63	134.99036	134.40151	135.42103	1.01952
PC18	11	40.422	7.4163	8.32	64.98322	64.22864	69.22887	5.00023
PC18	17	31.958	7.4163	7.81	61.38764	58.42704	62.28231	3.85526
PC18	19	36.670	7.4163	7.73	43.46057	42.68460	44.34706	1.66246
PC19	12	24.388	7.4248	8.09	56.41822	54.53122	56.90621	2.37498
PC19	12	25.938	7.4248	7.43	58.92540	58.59412	60.17437	1.58025
PC20	14	17.199	7.4380	8.05	29.12248	27.61014	32.47411	4.86397
