# Supplementary material for: Facial shape and allometry quantitative trait locus intervals in the Diversity Outbred mouse are enriched for known skeletal and facial development genes
Source: PLoS One. 2020 Jun 5;15(6):e0233377. doi: 10.1371/journal.pone.0233377 (PMC7274373; doi:10.1371/journal.pone.0233377)
Supplement: S2 Table — Full array of LOCO mean heritabilities over PCs 1–20. (PDF) [file pone.0233377.s004.pdf]

Chromosome	PC1	PC2	PC3	PC4	PC5	PC6	PC7	PC8	PC9	PC10	PC11	PC12	PC13	PC14	PC15	PC16	PC17	PC18	PC19	PC20
1	0.528	0.437	0.555	0.331	0.433	0.556	0.475	0.473	0.540	0.542	0.230	0.410	0.540	0.432	0.488	0.441	0.464	0.427	0.453	0.500
2	0.537	0.445	0.550	0.350	0.482	0.570	0.485	0.449	0.547	0.542	0.239	0.429	0.535	0.426	0.493	0.450	0.456	0.468	0.456	0.517
3	0.545	0.449	0.523	0.353	0.455	0.579	0.460	0.444	0.544	0.549	0.243	0.407	0.527	0.444	0.472	0.449	0.486	0.482	0.442	0.524
4	0.534	0.449	0.536	0.368	0.463	0.553	0.474	0.459	0.546	0.537	0.245	0.428	0.526	0.432	0.474	0.442	0.435	0.461	0.449	0.487
5	0.545	0.438	0.526	0.381	0.468	0.588	0.477	0.451	0.547	0.515	0.252	0.422	0.525	0.432	0.490	0.420	0.460	0.465	0.453	0.536
6	0.534	0.429	0.516	0.353	0.458	0.572	0.478	0.463	0.553	0.514	0.254	0.439	0.530	0.428	0.479	0.448	0.476	0.473	0.456	0.498
7	0.514	0.451	0.562	0.349	0.481	0.543	0.463	0.477	0.545	0.565	0.230	0.414	0.551	0.441	0.483	0.457	0.444	0.473	0.460	0.501
8	0.556	0.439	0.551	0.350	0.461	0.577	0.482	0.453	0.485	0.546	0.248	0.413	0.518	0.432	0.491	0.435	0.460	0.482	0.463	0.493
9	0.528	0.444	0.539	0.365	0.467	0.560	0.493	0.476	0.539	0.549	0.232	0.424	0.529	0.440	0.477	0.444	0.468	0.476	0.452	0.493
10	0.533	0.413	0.563	0.348	0.463	0.561	0.490	0.453	0.541	0.538	0.235	0.428	0.512	0.450	0.480	0.444	0.477	0.488	0.455	0.504
11	0.539	0.436	0.527	0.364	0.451	0.573	0.476	0.454	0.500	0.558	0.233	0.412	0.510	0.417	0.488	0.437	0.471	0.463	0.458	0.503
12	0.530	0.420	0.515	0.352	0.462	0.558	0.481	0.449	0.550	0.532	0.245	0.425	0.530	0.430	0.486	0.429	0.442	0.465	0.438	0.497
13	0.548	0.443	0.543	0.343	0.450	0.585	0.455	0.442	0.560	0.538	0.239	0.399	0.520	0.449	0.476	0.457	0.459	0.489	0.453	0.495
14	0.546	0.422	0.546	0.349	0.464	0.577	0.487	0.467	0.557	0.529	0.245	0.439	0.527	0.440	0.496	0.459	0.485	0.467	0.463	0.475
15	0.555	0.453	0.536	0.362	0.462	0.578	0.485	0.469	0.541	0.536	0.234	0.421	0.528	0.429	0.488	0.442	0.463	0.476	0.448	0.503
16	0.555	0.443	0.545	0.362	0.467	0.568	0.501	0.470	0.536	0.549	0.255	0.445	0.545	0.439	0.485	0.444	0.464	0.478	0.454	0.509
17	0.547	0.425	0.536	0.310	0.460	0.568	0.472	0.447	0.551	0.556	0.258	0.436	0.531	0.399	0.484	0.449	0.471	0.476	0.451	0.491
18	0.546	0.443	0.544	0.349	0.473	0.587	0.471	0.464	0.546	0.552	0.254	0.426	0.541	0.458	0.488	0.461	0.477	0.478	0.456	0.516
19	0.546	0.451	0.563	0.358	0.472	0.574	0.470	0.484	0.550	0.542	0.249	0.414	0.549	0.459	0.495	0.449	0.475	0.490	0.439	0.506
X	0.548	0.448	0.558	0.367	0.459	0.583	0.494	0.467	0.543	0.553	0.256	0.422	0.543	0.450	0.483	0.455	0.481	0.476	0.467	0.531
